# Regulatory Effects of Functional Soluble Dietary Fiber from *Saccharina japonica* Byproduct on the Liver of Obese Mice with Type 2 Diabetes Mellitus

**DOI:** 10.3390/md20020091

**Published:** 2022-01-21

**Authors:** Liping Zhang, Xixi Wang, Yingying He, Junhan Cao, Kai Wang, Huan Lin, Changfeng Qu, Jinlai Miao

**Affiliations:** 1 Department of Special Medicine, School of Basic Medicine, Qingdao University, Qingdao 266071, China; 15689487176@163.com; 2 Key Laboratory of Marine Eco-Environmental Science and Technology, First Institute of Oceanography, Ministry of Natural Resource, Qingdao 266061, China; WXi1173596132@163.com (X.W.); heyinging11@163.com (Y.H.); caojunhan1995@126.com (J.C.); wangkai9977@126.com (K.W.); linhuan202201@163.com (H.L.); 3 Laboratory for Marine Drugs and Bioproducts, Qingdao Pilot National Laboratory for Marine Science and Technology, Qingdao 266237, China

**Keywords:** *Saccharina japonica*, dietary fiber, PI3K/AKT, liver metabolomics

## Abstract

Though the relationship between dietary fiber and physical health has been investigated widely, the use of dietary fiber from marine plants has been investigated relatively rarely. The *Saccharina japonica* byproducts after the production of algin contain a large amount of insoluble polysaccharide, which will cause a waste of resources if ignored. Soluble dietary fiber (SDF)prepared from waste byproducts of *Saccharina japonica* by alkaline hydrolysis method for the first time had a wrinkled microscopic surface and low crystallinity, which not only significantly reduced liver index, serum levels of aspartate aminotransferase (AST) and alanine amiotransferase (ALT), and liver fat accumulation damage to the livers of obese diabetic mice, but also activated the PI3K/AKT signaling pathway to increase liver glycogen synthesis and glycolysis. By LC-MS/MS employing a Nexera UPLC tandem QE high-resolution mass spectrometer, the 6 potential biomarker metabolites were screened, namely glycerophosphocholine (GPC), phosphocholine (PCho), pantothenic acid, glutathione (GSH), oxidized glutathione (GSSG), and betaine; several pathways of these metabolites were associated with lipid metabolism, glycogen metabolism, and amino acid metabolism in the liver were observed. This study further provided a detailed insight into the mechanisms of SDF from *Saccharina japonica* byproducts in regulating the livers of obese mice with type 2 diabetes and laid a reliable foundation for the further development and utilization of *Saccharina japonica*.

## 1. Introduction

Obesity and type 2 diabetes mellitus (T2DM) are the most prominent chronic diseases worldwide, and they are associated with increased risk of several complicating conditions [1]. Obesity and diabetes are closely related. Currently, 387 million people worldwide are at risk of diabetes [2], of whom nearly 90% are obese [3], and 90% of obese patients have non-alcoholic fatty liver disease (NAFLD); furthermore, 80% of NAFLD patients have insulin resistance [4]. The reason for this is that insulin resistance can lead to triglyceride synthesis, peripheral adipose lipolysis, and excessive liver fat accumulation, which ultimately promotes the progression of NAFLD; therefore, obesity, NAFLD, and diabetes are a vicious circle, and the incidence is closely related [5]. The liver can be regarded as the chief organ in obesity-diabetes since it is the main organ for glucose and lipid metabolism, and abnormalities of gluconeogenesis and glycolysis in the liver are factors affecting diabetes patients with glucose disorders [6,7]. At present, the use of synthetic drugs is the most common clinical treatment method for obesity and diabetes, which likely induces a series of serious side effects, such as hypoglycemia, endocrine disorders, and drug resistance [8,9]. As a result, extracting active ingredients from plants to treat chronic diseases has become a heavily researched topic [10]. These ingredients are relatively effective, inexpensive, and almost free from side effects compared to synthetic drugs [11]. For example, polysaccharides from plants are utilized in attempting to treat obesity and diabetes [12].

The phosphatidylinositol 3-kinase (PI3K)/protein kinase B (PKB/AKT) signaling pathway is one of the main insulin signal transduction pathways, and it has a very close relationship with insulin resistance and T2DM [13]. When insulin binds to insulin receptors on the cell surface, the pathway is activated, and tyrosine residues on the insulin receptor substrate (IRS) are phosphorylated and migrate to the cell membrane, where their docking sites bind to the subunit of PI3K, thereby activating PI3K, which produces a second messenger phosphatidylinositol-(3,4,5)-trisphosphate (PIP3) on the plasma membrane, and then phosphorylates 3-phosphoinositide-dependent protein kinase-1 (PDK1) and activates AKT. AKT acts on a variety of downstream substrate receptors and regulates glucose uptake, glycogen synthesis, gluconeogenesis, and protein synthesis. Therefore, the PI3K/AKT pathway plays a crucial role in insulin signal transduction and insulin-mediated glucose metabolism [14]. It has been shown that resistant starch and pectin from fruit can improve insulin resistance and increase glycogen metabolism by activating the liver PI3K/AKT signaling pathway [15,16]. However, the activation of this pathway in liver tissue by the soluble dietary fiber of *Saccharina japonica* (formerly *Laminaria japonica*) has not been reported. Dietary fiber is an important polysaccharide, and has the function of alleviating hepatic insulin resistance, mainly by regulating glucose production and lipid accumulation effectively [17]. Meanwhile, it is also a kind of compound that protects the intestinal microecology and regulates and nourishes the intestinal flora [18,19]. The intestinal flora have a very strong metabolic capacity, which metabolizes dietary fiber into a variety of metabolites in the intestine. The hepatic portal vein system mainly receives intestinal blood and transfers metabolites to the liver, so that there is a close connection between the intestinal tract and the liver, forming the entero-hepatic axis. The composition of the intestinal flora may be involved in the pathogenesis of many chronic liver diseases, such as chronic hepatitis B, chronic hepatitis C, alcoholic liver disease, and non-alcoholic steatohepatitis [20].

*S. japonica* has rich nutritional value and is a natural health food, and its nutritional value has been extensively studied. For example, polysaccharides extracted from *S. japonica* can regulate intestinal microflora in high-fat-diet-induced diabetic mice [21], laminarin from *S. japonica* has good antioxidant capacity [22], and *S. japonica* fermented by *Lactobacillus brevis* FZU0713 has a potential function in lipid metabolism [23]. However, during the process of extracting the active ingredients from *S. japonica*, a large number of byproducts will be produced, which will result in energy waste and pollution if not treated in time. In previous studies, the byproduct after extracting algin from *S. japonica* was recovered and fully processed to obtain SDF with a purity of more than 82%, and the characterization of SDF was deeply studied [24]. 

In the current research, the aim was to explore the underlying molecular mechanism of SDF in alleviating diabetic symptoms via related genes and conduct a characteristics analysis of its physiological and biochemical effects on the liver. For this purpose, the normal control group, model control group, and model SDF group were given intragastric administration, and then the changes in liver metabolites and gene expression were observed. This is the first report to show that *S. japonica* byproducts can regulate diabetes at the liver level and provide theoretical information for the application of SDF in functional foods.

## 2. Results and Discussion

### 2.1. Characterization of SDF

**Figure 1:**
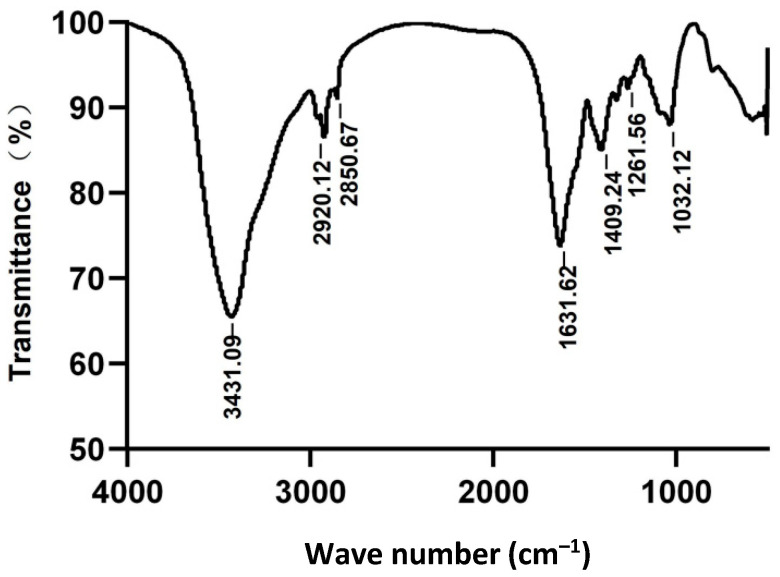
The FT-IR spectra of SDF and the Y-axis is transmittance, and the X-axis is wave number.

The Fourier transform infrared (FT-IR) spectra of SDF in Figure 1 revealed that SDF was a typical sugar spectrum from the overall spectrum [25]. The strongest and rounder peak appeared at 3431.09 cm^−1^, suggesting that there were indications of O-H stretching vibration and intermolecular and intramolecular hydrogen bonding in SDF [26], which could increase its hydrophilicity. The peaks of 2920.12 and 2850.67 cm^−1^ indicated that there is C-H stretching vibration in the methyl group [27]. The detection of peak table at 1409.24 cm^−1^ corresponded to C-H bending vibration, and the peak at 1032.12 to 1261.56 cm^−1^ was assigned to C-O stretching vibration. A peak at 580.93 cm^−1^ was possibly due to the blending vibration of β-C-H [26]. The peak area of O-H was the largest, indicating that there were more intramolecular hydrogen bonds, which increased the hydrophilicity of soluble *S. japonica* dietary fiber.

**Figure 2:**
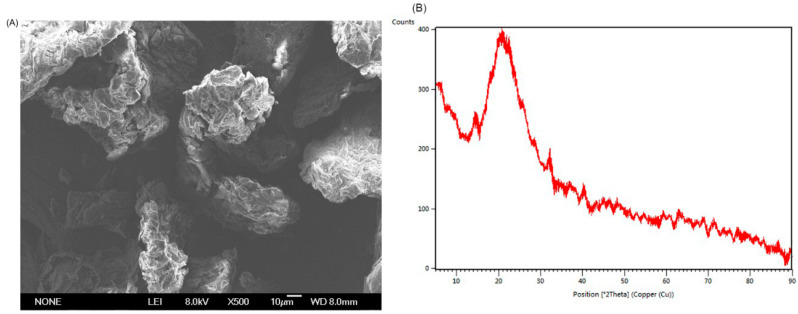
The microscopic topography of SDF at magnification 500× (**A**). The XRD patterns of SDF at 2θ angle (**B**).

The scanning electron microscopy (SEM) is an important method for studying the microstructure of SDF [26]. The SEM image of SDF 500× in Figure 2A displayed that SDF had an uneven and wrinkled surface, which was conducive to the absorption of glucose, cholesterol, and other harmful substances. The wave drapes of SDF could enlarge its surface area and expose more polar groups such as hydroxyl and carboxyl groups, as well as other sites where water binds, promoting the adsorption and binding of water [28]. Moreover, the SDF had a little crystalline structure (5.67%) and most of the non-crystalline structure in the X-Ray Diffraction (XRD) spectrum (Figure 2B). The higher the crystallinity, the better the tensile strength and hardness, while the lower the crystallinity, the more improve the water swelling, oil absorption capacity, and other functional characteristics [29]. According to SDF characterization of the source of *S. japonica* byproducts, the SDF had the potential to be used as functional food or a food additive.

### 2.2. Effects of SDF on the Liver in db/db Mice

**Figure 3:**
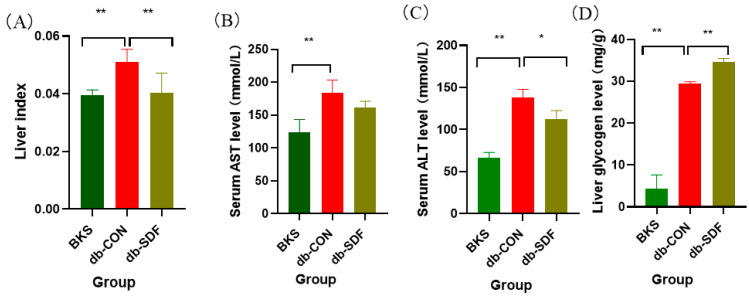
Effects of SDF on liver, the liver index (**A**), the serum content of AST (**B**), the serum content of ALT (**C**), the liver glycogen (**D**). (*n* = 5, * *p* < 0.05, ** *p* < 0.01).

The liver index of the model control group (db-CON) mice was significantly heavier than that of the normal control group (BKS) mice, as shown in Figure 3A. Consistent with our hypothesis, the liver index was effectively in remission after 5 weeks of SDF treatment (*p* < 0.01), so the liver hypertrophy due to obesity was relieved by SDF intervention. ALT and AST are primarily observed in liver cells, and they are released into the blood when liver function is impaired, for example, abnormal glucose and lipid metabolism damaged liver tissue [30]. As shown in Figure 3B,C, ALT, and AST levels were elevated significantly (*p* < 0.01) in db-CON mice relative to the BKS group, whereas SDF intervention effectively reversed this severe phenomenon and reduced AST and ALT by 12.19% ± 0.04 and 18.32% ± 1.34 (*p* < 0.05), respectively, which indicated that SDF could ameliorate liver injury caused by obesity and diabetes. The liver is very sensitive to insulin and can regulate glycogen production, which plays a key role in the regulation of glucose homeostasis in the body [31]. As shown in Figure 3D, after 5 weeks of intervention, SDF significantly increased the glycogen content in the liver compared with the db-CON group (*p* < 0.01), which reduced free glucose in the blood vessels through glycogen synthesis. It is worth mentioning that the liver glycogen content in the db-CON group was also significantly higher than that in the BKS group (*p* < 0.01), and the reason might be that the content of liver glycogen in db/db mice was originally much higher than that in normal mice.

### 2.3. Effects of SDF on Histopathology

**Figure 4:**
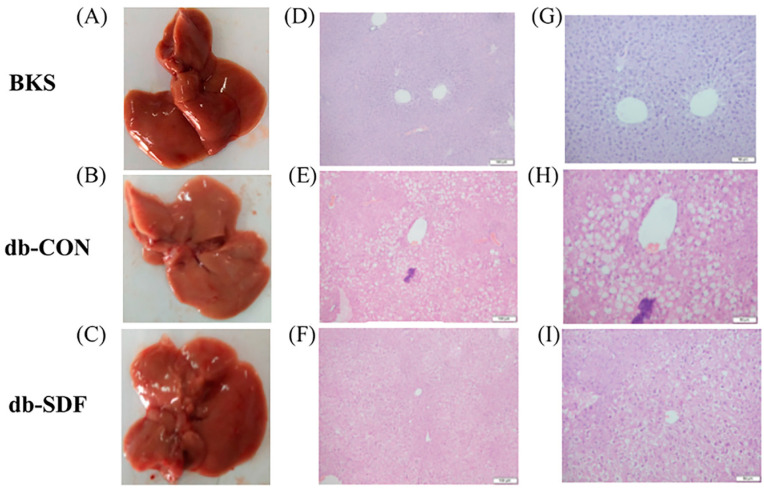
H&E pathological sections of liver tissue ((**A**–**C**) were representative macroscopic images, (**D**–**F**) were H&E staining sections 200×, (**G**–**I**) were H&E staining sections 400×).

Heterotopic fat accumulation in obese individuals is the main cause of NAFLD, which is also a major cause of liver hypertrophy and hepatic steatosis [32]. The morphology and structure of liver tissue were shown, demonstrating that the livers of the BKS mice had a smooth surface with cells arranged regularly and no visible accumulation of lipid droplets in H&E-stained tissue sections (Figure 4A,D,G). In contrast, there were numerous lipid droplets accumulated in the liver tissue sections, and the hepatocytes were irregularly arranged in the db-CON group (Figure 4B,E,H). After treatment with SDF for 5 weeks, the size of the lipid droplets decreased and returned to the size observed for normal mice, the hepatocytes were arranged tightly, and the surface of the liver also had a restored luster compared with the db-CON group (Figure 4C,F,I). There is evidence that the improvement of liver histology (steatosis, inflammation, and fibrosis) is related to a reasonable diet, and the formulated food that contains dietary fiber may improve these symptoms by altering its metabolites [33]. These results suggested that dietary supplements may be a potential treatment for NAFLD patients.

### 2.4. Effects of SDF on PI3K/AKT Insulin Signaling Pathway

**Figure 5:**
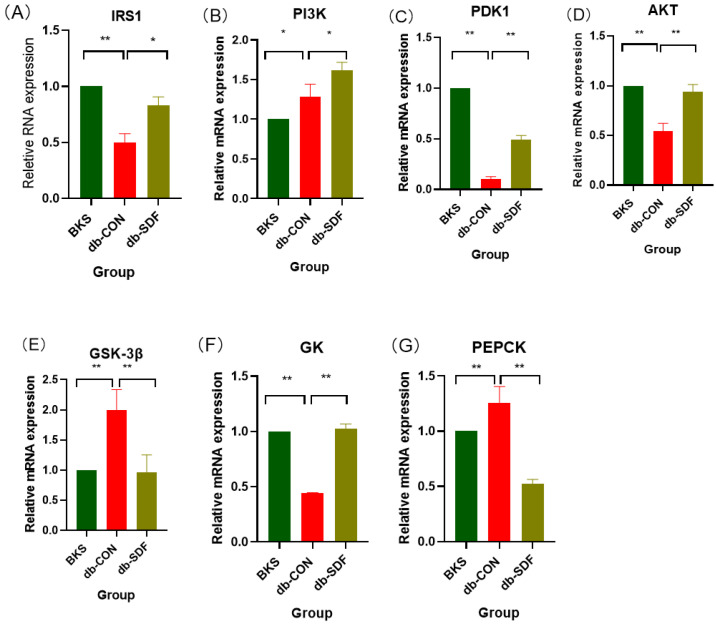
The relative mRNA expression of the PI3K/AKT insulin signaling pathway in the liver, IRS1 (**A**), PI3K (**B**), PDK1 (**C**), AKT (**D**), GSK-3β (**E**), GK (**F**), PEPCK (**G**). (*n* = 5, * *p* < 0.05, ** *p* < 0.01).

Insulin can phosphorylate IRS1 and activate PI3K, and then activate PDK1, which is a key mediator for activating AKT. When AKT is activated, all key enzyme genes in the PI3K/AKT signaling pathway in the liver are activated, which can increase glucose uptake from the periphery of cells and stabilize the glucose stability of the body [34]. The insulin signaling pathway was impaired in obese diabetic mice, so the relative expression levels of IRS1, PDK1, and AKT were significantly decreased compared with BKS mice (*p* < 0.01) (Figure 5A,C,D). Curiously, PI3K expression was significantly increased in Figure 5B (*p* < 0.05), which might be related to its negative feedback regulation, which needed to be discussed in the future. However, after 5 weeks of SDF intervention, the relative expression levels of IRS1 and PI3K were significantly higher than those of the db-CON group (*p* < 0.05), and the expression levels of PDK1 and AKT were significantly increased (*p* < 0.01). When the PI3K/AKT pathway was activated, it regulated downstream glucose metabolism and reduced glucose output by increasing glycogen synthesis [31,35]. GSK-3β can regulate the activity of related enzymes in the glycolysis pathway by phosphorylating liver glycogen synthase, and the decrease of GSK-3β relative expression can improve the activity of liver glycogen synthase, increase liver glycogen synthesis, and reduce liver glycogen output. Glucokinase (GK) is the first rate-limiting enzyme in the glycolysis pathway and can catalyze the glucose-6-phosphatase catalytic (G6PC), which is also the premise of liver glycogen synthesis [36]. Moreover, when the body is in a state of hyperglycemia, GK can increase the uptake of glucose by the liver, and also regulate the synthesis and secretion of insulin by β cells. Phosphoenolpyruvate carboxykinase (PEPCK), the key enzyme in gluconeogenesis, was also detected. This is because reducing PEPCK enzyme activity is beneficial to the gluconeogenesis process, reducing the output of blood sugar, thus achieving the effect of reducing blood sugar [15]. The expression levels of GSK-3β and PEPCK in the db-CON group were significantly higher than those in the BKS group, while GK was significantly decreased (*p* < 0.01). As expected, SDF significantly reduced the expression of GSK-3β and PEPCK (*p* < 0.01) and increased the expression of GK (*p* < 0.01) (Figure 5E–G); therefore, SDF could increase liver glycogen synthesis and decrease gluconeogenesis in db/db mice, which is consistent with the trend of liver glycogen results.

### 2.5. Metabolomic Analysis

#### 2.5.1. Quality Control

**Figure 6:**
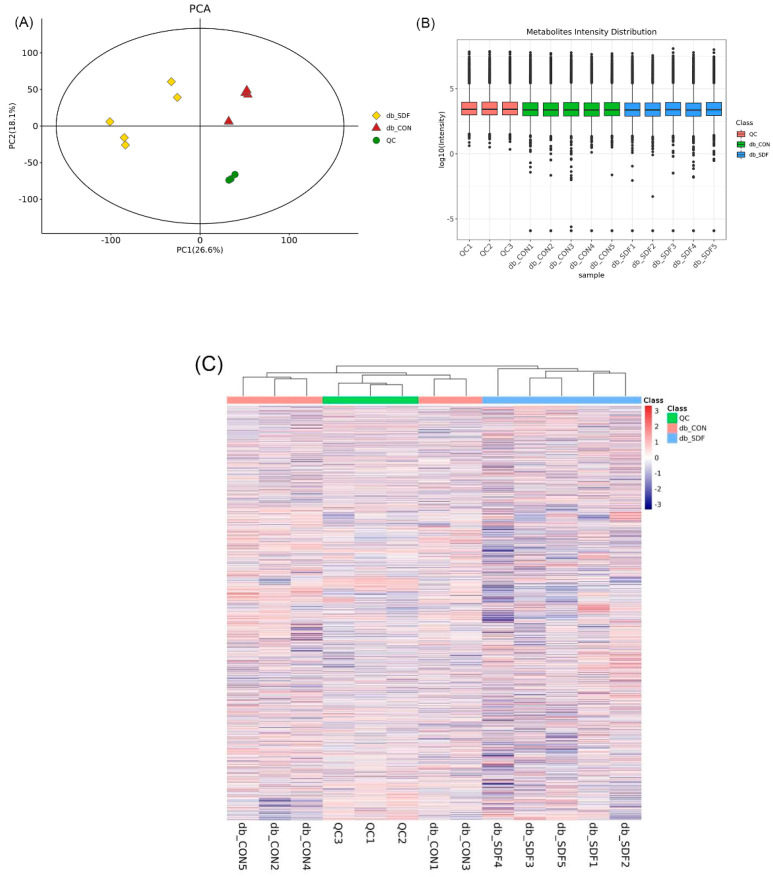
QC samples on the PCA (**A**), the metabolite strength boxplot of all samples (Y-axis was the log_10_ value of mass spectrum strength) (**B**), the cluster heat map of all samples (**C**), different colors represented different groups, as shown in the top right sides of the figures (*n* = 5).

Mass spectrometry system stability was assessed and evaluated by the quality control (QC) samples inserted in the sequence (QC, *n* = 3, experiment sample, *n* = 5). After 7 cycles of cross experiments, the ion peak of relative standard deviation (RSD) > 0.4 in the QC group was deleted, and QC samples were clustered together closely in the principal component analysis score plot (PCA) (Figure 6A). In the boxplot (Figure 6B), QC sample metabolite strength was aggregated, and no dispersion was apparent. To more intuitively show the relationship between QC samples and other samples, as well as the stability among QC samples, all metabolite expressions were hierarchically clustered (Figure 6C). These findings, combined with the above results, demonstrated that the metabolomic method was stable and reproducible.

#### 2.5.2. Multivariate and Univariate Data Analysis

**Figure 7:**
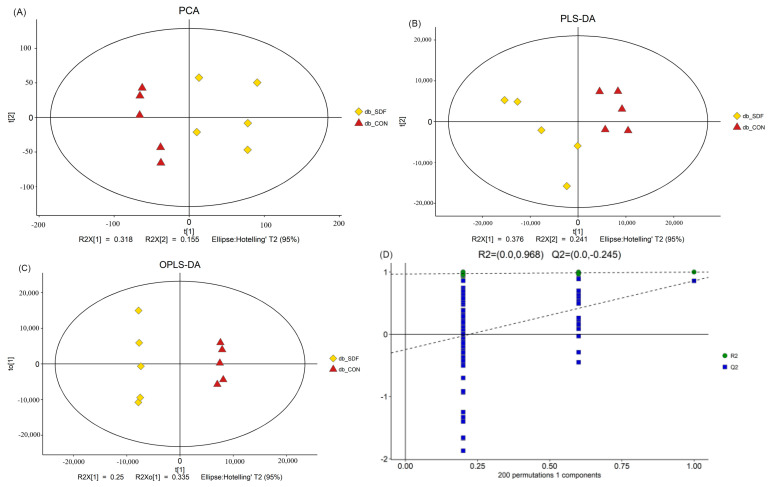

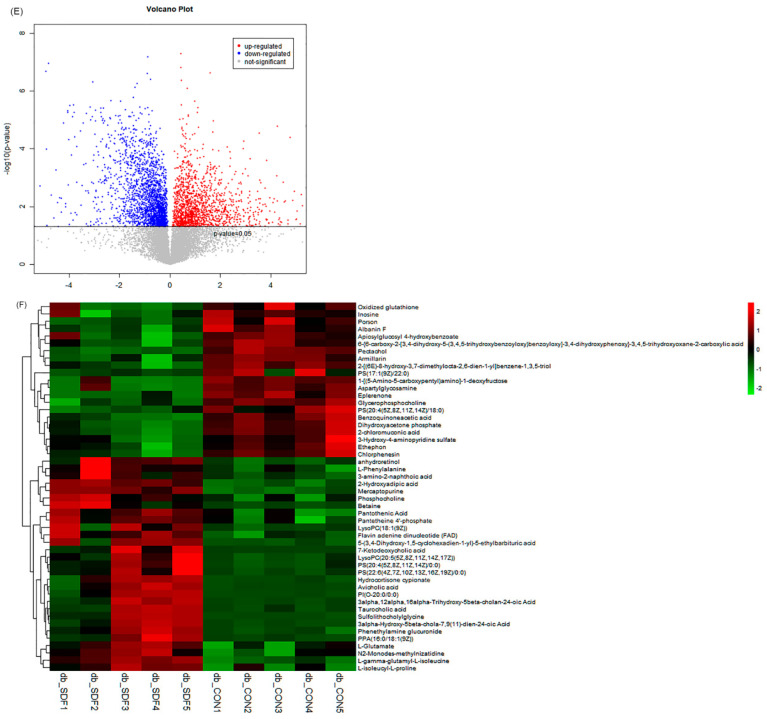
PCA (**A**), PLS-DA (**B**), OPLS-DA (**C**) and validation plots of OPLS-DA (**D**) in db-SD /db-CON, the Hotelling’s T^2^ were 95%, color red represented db-CON, yellow represented db-SDF, linear regression was conducted with R^2^Y and Q^2^Y of the original model, and the intercept values of the regression line and Y-axis obtained were R^2^ and Q^2^, respectively, which were used to measure whether the model was over-fitted. In the volcano plot of the db- SDF/db- CON group (**E**), the *p*-value was 0.05, and the red origin represented the significantly up-regulated differential metabolites, while the blue origin represented the significantly down-regulated differential metabolites and the gray point represented the insignificant differential metabolites. The differential metabolite heat map of db-SDF/db-CON (**F**) and the degree of change were marked with red and green, representing up-regulation and down-regulation, respectively.

The population distribution among samples and the stability of the whole analysis process were analyzed by PCA (Figure 7A). The overall difference of metabolic profile between each group and the different metabolites between groups were determined by supervised partial least square discrimination analysis (PLS-DA) (Figure 7B) and orthogonal partial least square discrimination analysis (OPLS-DA) (Figure 7C). The clusters of the model SDF group (db-SDF) and the db-CON group were separated from each other, indicating that SDF treatment could change the liver metabolites compared with the db-CON group, and the changed metabolites in liver will affect multiple metabolic networks, which play an important role in maintaining body metabolism [37]. To prevent the overfitting of the model, 7-fold cross-validation and response permutation testing (RPT) were utilized to investigate the quality of the model (Figure 7D), and all the model parameters were presented (Table S2). All of the R^2^X > 0.4, R^2^ values were close to 1, and Q^2^ < 0, therefore, these parameters indicated that the model avoided over-fitting and was considered good [38]. The univariate analysis mainly focuses on the description of univariate and statistical inference, which primarily includes interval estimation and statistical hypothesis testing. A volcano plot was used to visualize the *p*-value and the fold changes value, which was used for screening the differential metabolites. There were 12,609 total differential metabolites, including 1511 significantly up-regulated differential metabolites, 2213 significantly down-regulated differential metabolites, and 8885 insignificantly differential metabolites (Figure 7E). Therefore, functional metabolite molecules present in the significantly altered metabolites are helpful to explore the regulatory mechanism of SDF on the liver.

#### 2.5.3. Differential Metabolites Analysis

**Figure 8:**
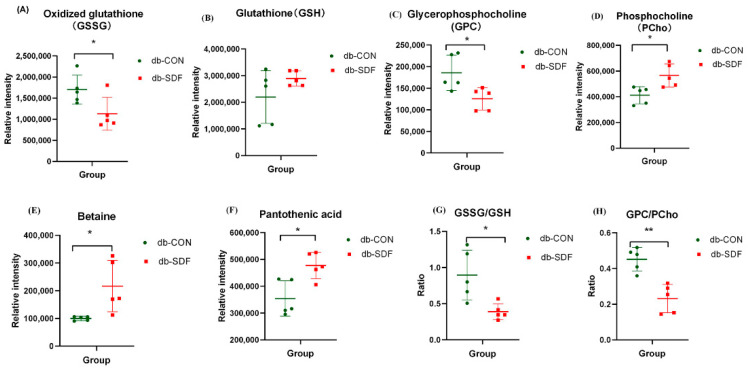
Differential metabolites associated with oxidative stress and inflammation, oxidized glutathione (GSSG) (**A**), glutathione (GSH) (**B**), glycerophosphocholine (GPC) (**C**), phosphocholine (PCho) (**D**), pantothenic acid (**E**), betaine (**F**), GSSG/GSH (**G**), GPC/PCho (**H**) (*n* = 5, * *p* < 0.05, ** *p* < 0.01).

From the results of multivariate and univariate analysis, differential metabolites between groups were screened, and significant differential metabolites were investigated. In the variable importance in projection (VIP) of OPLS-DA analysis, a VIP > 1 was considered to indicate differential metabolites. To further verify the significance of the differential metabolites, the significance was judged by *t*-test method, and the *p*-value < 0.05 was of significance. The results of the analysis showed that the db-SDF group had 205 significantly different metabolites (125 down-regulated and 80 up-regulated) compared with the db-CON group. The top 50 significantly different metabolites were identified (Figure 7F), and the correlation of the top 50 differential metabolites used the Pearson correlation coefficient for analysis (Figure S1). The 6 metabolites associated with obesity, high glucose, inflammation, oxidative stress, and cancer were screened and analyzed for significance in Figure 8, being GSH, GSSG, GPC, PCho, betaine, and pantothenic acid, respectively.

The db/db mice are characteristic of chronic hyperglycemia, hyperlipidemia, and insulin resistance, which increases reactive oxygen species (ROS) production and decreases the activity of antioxidant enzymes [39]. Increased ROS production leads to neutrophil infiltration and further produces widespread inflammation, which poses a threat to the immune system and leads to other complications [40]. GSH is a tripeptide, consisting of cysteine, glutamate, and glycine, which is an important antioxidant and free radical scavenger in the body. GSSG is the oxidative dimer form of glutathione, a naturally occurring tripeptide, which is observed in antioxidant damage. A lower GSSG concentration was observed in liver metabolites of db-SDF/db-CON (ID = 0.93_611.1446 *m*/*z*, *m*/*z* = 611.1446301, C_20_H_32_N_6_O_12_S_2_, VIP = 6.05, down-regulated 0.66 times, *p* < 0.05) (Figure 8A), and the GSH level in the liver did not show significant changes (ID = 0.92_307.0837n, *m*/*z* = 308.0909559, C_10_H_17_N_3_O_6_S) (Figure 8B). A higher ratio of GSSG to GSH had been observed in obesity and diabetes patients [41], and the ratio of GSSG to GSH in the mice receiving the SDF supplementation was significantly lower than in the db-CON mice (*p* < 0.05) (Figure 8G). These results indicated that the suppressive effects of SDF on oxidative stress and liver injury could be obtained by mediating GSG and GSSG concentration in db/db mice.

GPC is composed of choline, glycerol and phosphate, and is the source of choline for the synthesis of acetylcholine and phosphatidylcholine, which is directly associated with body weight and is the indicator of several types of cancers [42]. The GPC (ID = 9.89_508.3766 *m*/*z*, *m*/*z* = 508.3765518, C_40_H_76_NO_8_P, VIP = 2.09, down-regulated 0.67 times, *p* < 0.05) and PCho (ID = 0.92_184.0734 *m*/*z*, *m*/*z* = 184.0733593, C_5_H_14_NO_4_P, VIP = 3.45, up-regulated 1.37 times, *p* < 0.05) were significantly increased and decreased compared to the db-CON group after 5 weeks of SDF intervention, respectively (Figure 8C,D), and the ratio of GPC/PCho was significantly different to a high degree of certainty (*p* < 0.01) (Figure 8H). A high ratio of GPC/PCho is generally observed in obesity, and GPC metabolites are also associated with the risk of cardiovascular and cerebrovascular diseases and the development of insulin resistance [43]. Therefore, the level of GPC and the ratio of GPC/PCho in db-SDF/db-CON suggested that SDF could reduce the associated risk of concurrent disease induced by obesity and high glucose.

Betaine is a quaternary amine alkaloid, a glycine trimethylamine salt, and a methyl donor for animal nutrition. Differential metabolite analysis showed that the betaine concentration was highly significant in db-SDF/db-CON (ID = 0.87_117.0790n, *m*/*z* = 156.0421476, C_5_H_11_NO_2_, VIP = 2.94, up-regulated 2.16 times, *p* < 0.05) (Figure 8E). In addition, accumulating evidence had demonstrated that betaine could counteract oxidative stress and inflammation and alleviate several diseases, including obesity, diabetes, cancer, and Alzheimer’s disease [44]. The concentration of betaine is also positively related to the level of methionine, which plays an essential role in antioxidation by chelation and the synthesis of glutathione in the liver [45,46]. Furthermore, betaine notably activates adenosine 5‘-monophosphate (AMP)-activated protein kinase phosphorylation, which promotes glucose transporter type 4 (GLUT-4) to take up glucose and suppresses acetyl-CoA carboxylase activity as well as sterol regulatory element-binding protein-1c and fatty acid synthase expression to attenuate fat accumulation. Therefore, the level of betaine in the liver was closely associated with the effects of SDF on glucose and lipid metabolism [47,48].

Pantothenic acid (vitamin B5), a B vitamin converted from pantetheine, participates in lipid hydrolysis in the body [49], and is also the main component of coenzyme a (CoA), cysteine and the production of adenosine triphosphate (ATP). Moreover, it may improve glucose and lipid metabolism by affecting the tricarboxylic acid cycle [50,51]. The concentration of pantothenic acid was increased by SDF treatment (ID = 2.46_219.1108n, *m*/*z* = 220.1180329, C_9_H_17_NO_5_, VIP = 3.11, up-regulated 1.35 times, *p* < 0.05) (Figure 8F), therefore, SDF could reduce fat accumulation and glucose metabolism in the liver by increasing the content of pantothenic acid in db/db mice.

#### 2.5.4. KEGG Pathway Analysis

**Figure 9:**
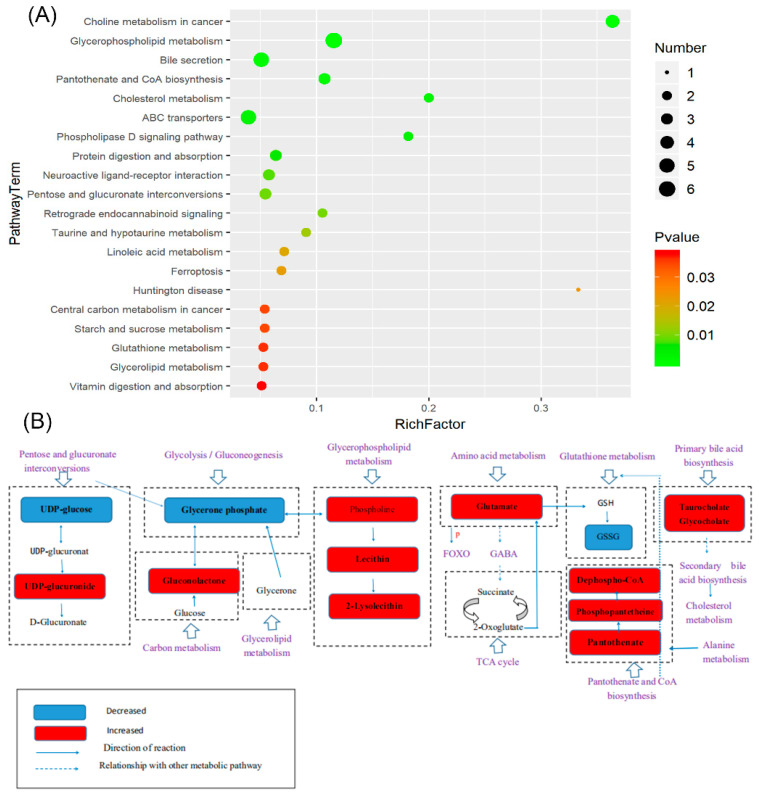
The top 20 enriched pathways and the X-axis was the rich factor that was the ratio of significantly different number of metabolites to the total number of metabolites in this pathway; the larger the black circle, the higher the number of significantly different metabolites in the pathway. The Y-axis was the name of the metabolic pathway; the color from red to green indicates that the *p*-value decreases successively (**A**). The schematic diagram of the relationship between metabolites and potential metabolic pathways is (**B**).

A metabolic pathway enrichment analysis of differential metabolites was performed based on the KEGG database (https://www.kegg.jp/, accessed on 16 February 2021). In the top 20 pathway terms (Figure 9A), the enrichment factor was determined to be directly proportional to the level of enrichment in the pathway, and the enrichment levels of all the significant metabolic pathways were listed (*p* < 0.05) (Table S3), where the glycerolipid metabolism, glutathione metabolism, starch and sucrose metabolism, central carbon metabolism in cancer, taurine and hypotaurine metabolism, pentose and glucuronate interconversions, protein digestion and absorption, ABC transporters, cholesterol metabolism, pantothenate and CoA biosynthesis, bile secretion, glycerophospholipid metabolism, and vitamin digestion and absorption pathways played crucial roles in the metabolic processes of obesity and diabetes. It is well known that the liver of obese and diabetic patients suffers great damage, so the KEGG pathway analysis of the liver with SDF intervention was mainly enriched regarding liver protection. The glycerolipid metabolism is an important targeted pathway for regulating NAFLD, wherein the glycerolipid/free fatty acid cycle in glycerolipid metabolism can prevent hepatic steatosis and inflammation [52,53]. Glutathione metabolism plays an important role in antioxidant protection, nutrient metabolism, cytokine production, and immune response [54,55]. Starch and sucrose metabolism are uniquely related to the relief of liver damage [56], and pantothenate and CoA biosynthesis is related to oxidative stress [57]. In addition, the ABC transporter is the key to transport compounds in the liver and is abundant, which plays a role in immune cell-mediated inflammation because of their ability to transport prostaglandins and leukotrienes [58,59]. The association between metabolic pathways and differential metabolites was presented (Figure 9B). These data combined with the patent result (patent number: ZL 201911164157. 7) suggested that SDF could not only effectively regulate intestinal flora and intestinal health, but also protect the liver and further improve obesity and diabetes through the hepatic-intestinal axis.

## 3. Materials and Methods

### 3.1. Reagents and Materials

All chemicals and solvents were analytical-grade or HPLC-grade. The *S. japonica* byproducts used to produce soluble dietary fiber were provided by Yantai Intercontinental Marine Biotechnology Co., Ltd. (Yantai, Shandong, China), which was the algae debris after the production of algin. The SDF (purity > 82%) was extracted by the First Institute of Oceanography, Ministry of Natural Resources (Qingdao, China). The Trizol Reagent, EasyScript^®^ One-Step gDNA Removal and cDNA Synthesis SuperMix, and perfectStart^®^ Green qPCR SuperMix (+Dye I) were purchased from Beijing TransGen Biotech Co., Ltd. (Beijing, China). The glycogen content and ALT and AST determination kit were purchased from Beijing Solarbio Science & Technology Co., Ltd. (Beijing, China). Water, methanol, acetonitrile, and formic acid were purchased from CNW Technologies GmbH (Düsseldorf, Germany). L-2-chlorophenyl alanine was obtained from Shanghai Hengchuang Biotechnology Co., Ltd. (Shanghai, China).

### 3.2. Methods

#### 3.2.1. Preparation of SDF from *S. japonica* Byproduct

The preparation of SDF from *S. japonica* byproducts was described briefly [24]. By soaking the *S. japonica* byproduct with 5 M sodium hydroxide solution (material to solvent 1:30), treating it at high temperature (90 °C) for several hours, centrifuging it with a centrifuge, and then overnight dialyzing supernatant with 1500 D dialysis bag, the frozen-vacuum-dried powder of dialyzed SDF solution was prepared. The isolated yields were 20–25%, and the purity of the prepared products attained 82 ± 1%.

#### 3.2.2. Characterization of SDF

The FT-IR spectra of the SDF sample were detected by an ATR accessory (Vertex 70, Bruker, Germany). The 2 mg SDF and 100 mg KBr (spectral grade) were ground and pressed into a transparent sheet after grinding. The background was blank KBr, the scanning time was 20 times, the resolution was 4 cm^−1^, and the scanning range was 4000–400 cm^−1^.

The XRD (Bruker D8 ADVANCE, Germany) was used to detect the morphology of the SDF. The scanning range of 2θ was 5° to 90° and the stride length was 4°/min. Crystallinity was calculated using MDI Jade v6.5 software.

SEM of SDF was observed using SU8020 field emission scanning electron microscope (Japan) with ion sputtering coating method. The voltage was 5.0 Kv, and the magnification was 500×.

#### 3.2.3. Animal Treatments and Sample Collection

The SPF grade db/db mice and wild-type BKS mice (female, aged 6 weeks) were provided by Nanjing Biomedical Research Institute of Nanjing University (Nanjing, China). The mice were housed in a stable environment, where the temperature was 22 ± 2 °C; relative humidity was 50% to 60%, which was provided *ad libitum*; and a 12-h light/dark cycle was employed. After 1 week of acclimatization, animals were randomly divided into three groups (*n* = 7): the normal control group (BKS, intragastric gavage with vehicle), the model control group (db-CON, intragastric gavage vehicle), and the model SDF group (db-SDF, intragastric gavage 5 g/kg body weight SDF). In this patent, the extraction method and dosage of SDF were described (patent number: ZL 201911164157. 7). At the end of the experiment, body weight and total liver weight were recorded to calculate the liver index according to the method [60]. The 0.1 g of the liver was cut, wrapped in tin foil, and immediately frozen in liquid nitrogen for liver glycogen detection and quantitative real-time PCR (qRT-PCR). All animal experimental procedures were approved by the Institutional Animal Care and Use Committee, Ocean University of China (Certificate no. SYXK20120014).

#### 3.2.4. The Serum ALT, Serum AST, Liver Glycogen Detection and Histological Morphology Examinations

The blood samples were collected and left standing for 30 min, then centrifuged at 3000× *g* for 10 min, and the serum was separated for serum ALT and AST analysis according to previous studies and biochemical assay kit instructions [61]. The amount of glycogen from 0.1–0.2 g liver samples was determined with a Glycogen Assay Kit (Solarbio), which was normalized by weight.

Liver samples, after being properly sized (1 cm × 1 cm), were placed in 10% neutral formaldehyde for >24 h and subjected to gradient alcohol dehydration (eluting with 95% ethanol for 2 times, 5 min each time, and adding anhydrous ethanol for 2 times, 5 min each time), paraffin embedding, sectioning (5 μm—thick sections), and hematoxylin and eosin (H&E) staining. Finally, tissue was observed under high magnification and photographed (Olympus).

#### 3.2.5. Gene Expression Analysis by qRT-PCR

0.1 g liver tissue was taken out at −80 °C and then put into a mortar with liquid nitrogen for full grinding. Total RNA was extracted by the Trizol method, RNA integrity was verified by gel electrophoresis, and the concentration of RNA and the ratio of A260/280 were measured by NanoDrop One Microvolume UV-Vis Spectrophotometer (Thermo Fisher Scientific, Wilmington, DE, USA). Total RNA reverse transcription to cDNA was conducted following the EasyScript^®^ One-step gDNA Removal and cDNA Synthesis SuperMix kit. The qRT-PCR was performed with the perfect Start ^®^ Green qPCR SuperMix (+Dye I) kit, and the PCR cycling conditions were as follows: 95 °C for 30 s, and then 40 cycles (95 °C for 5 s, 55 °C for 15 s, 72 °C for 10 s), 95 °C for 60 s, 57 °C for 30 s, and 95 °C for 30 s. The gene sequences of IRS1, PI3K, PDK1, AKT, GSK-3β, GK, and PEPCK were downloaded from NCBI and the qRT-PCR primers were synthesized and purchased from Sangon Biotech (Shanghai, China) Co., Ltd., listed in Table S1. Quantitative results were obtained using the 2^−ΔΔCt^ method, and the calculation formula was based on previous studies [62].

#### 3.2.6. Metabolomics Analysis

After being accurately weighed, 30 mg of liver was transferred to a 1.5 mL Eppendorf tube with two small steel balls. A total of 20 μL internal standard (2-chloro-l-phenylalanine in methanol, 0.3 mg/mL and Lyso pC17:0 in methanol, 0.01 mg/mL) and 400 μL methanol/water (4/1, *v*/*v*) were added to each sample. Samples were stored at −20 °C for precooling and then ground at 60 Hz for 2 min, respectively, followed by ultrasonication in an ice bath for 10 min and storage at −20 °C for 20 min. Next, the extract was centrifuged for 10 min (13,000 rpm, 4 °C), and 300 μL of supernatant was dried by centrifugal vacuum freezing in a brown glass vial before being reconstituted by 400 μL of methanol/water (1/4, *v*/*v*). This was then vortexed for 30 s, sonicated for 2 min, and centrifuged for 5 min (13,000 rpm, 4 °C). Next, 150 μL of supernatants from each sample were collected using crystal syringes, filtered through 0.22 μm microfilters, and transferred to LC vials for LC-MS/MS analysis. Quality control samples were prepared by mixing equal volumes of extracts from all samples, and each QC had the same volume as the sample. All extraction reagents were precooled at −20 °C before using.

UPLC: The chromatographic column was ACQUITY UPLC BEH C_18_ (100 mm × 2.1 mm, 7 µm, Waters Corporation, Milford, CT, USA) with a column temperature of 45 °C. Mobile phase: A-water (containing 0.1% formic acid, *v*/*v*), B-acetonitrile (containing 0.1% formic acid, *v*/*v*); flow rate: 0.35 mL/min; the injection sample volume was 10 μL; gradient elution was as follows: 0.01 min to 3 min, 95% A and 5% B; 3 min, 70% A and 30% B; 7 min, 40% A and 60% B; 9 min, 10% A and 90% B; 11–15 min, 100% B; and 15 min, 95% A and 5% B. During the analysis, all samples were stored at 4 °C.

Mass spectrometry: A Q Exactive quadrupole orbitrap mass spectrometer was equipped with a heated electrospray ionization (ESI) source (Thermo Fisher Scientific, Waltham, MA, USA), and the sample mass spectrum signal acquisition adopted positive and negative ion scanning mode. The parameters of mass spectrometry were as follows: the mass scan range, 60–900 (+) and 60–900 (−); resolution (full scan), 70,000 (+) and 70,000 (−); resolution (HCD MS/MS scans), 17,500 (+) and 17,500 (−); spray Voltage (V), 3500 (+) and 3100 (−); sheath gas flow rate (Arb), 30 (+) and 30 (−); auxiliary gas flow rate (Arb), 10 (+) and 10 (−); capillary temperature (°C), 320 (+) and 320 (−).

The row dates of LC-MS/MS were collected by UNIFI 1.8.1 software and analyzed by the progenesis QI v2.3 soft (Nonlinear Dynamics, Newcastle, UK), which performed the baseline filtering, peak identification, integration, retention time correction, peak alignment, and normalization; its main parameters were the following: precursor tolerance, 5 ppm; product tolerance, 10 ppm; production threshold, 5%; retention time (RT) tolerance, 0.02 min; minimum intensity, 15%. The identification of the compound was based on accurate mass, secondary fragmentation, and isotope distribution; moreover, the qualitative identification used the Human Metabolome Database (HMDB) and the Lipidmaps (v2.3) and METLIN databases.

For the extracted data, ion peaks with missing values (0 value) > 50% were deleted and replaced with half of the minimum value, and the results were qualitatively screened by obtaining compounds for subsequent analysis.

### 3.3. Statistical Analysis

The results were expressed as the mean ± standard deviation (SD) and analyzed by Student’s *t*-test for two groups. They were also analyzed using one-way analysis of variance (ANOVA) for three groups with Tukey. The analysis of differential metabolites was based on variable weight value (variable importance of projection, VIP) that was obtained from the OPLS-DA model, and *p*-values from a two-tailed Student’s *t*-test on the normalized peak areas, where VIP > 1 and *p* < 0.05 were considered to differential metabolites.

## 4. Conclusions

The *S. japonica* byproduct after the production of algin contains a large amount of insoluble cellulose from the cell wall, which is a high-quality raw material for the extraction of SDF. In this study, SDF was obtained through the alkaline hydrolysis process and had a wrinkled microscopic surface and low crystallinity. We further investigated the mechanism of SDF regulating the liver on obese mice with T2DM by alleviating liver adipose accumulation lesion, activating the PI3K/AKT signaling pathway, and changing the liver metabolites. There were 6 potential biomarker metabolites, namely, GPC, PCho, pantothenic acid, GSH, GSSG, and betaine, which were identified by combining multivariate with univariate analysis. These results further demonstrated the underlying metabolic pathways of SDF for reducing blood glucose by KEGG pathway analysis, and many pathways were associated with lipid metabolism, glycogen metabolism, and amino acid metabolism in the liver. These results may establish a foundation for future efforts to reduce the waste of *S. japonica* resources and represent an important advance in understanding the beneficial effects of SDF on obesity and diabetes.

## Data Availability

Not applicable.

## Supplementary Materials

The following supporting information can be downloaded at: https://www.mdpi.com/article/10.3390/md20020091/s1, Figure S1: The correlation of the top 50 differential metabolites used the Pearson correlation coefficient for analysis; Table S1: Primer sequences for qRT-PCR; Table S2: Parameters for the assessment of these models; Table S3: Significantly metabolite pathway enrichment.

## Abbreviations

SDF, soluble dietary fiber; T2DM, diabetes mellitus type 2; FT-IR, Fourier transform infrared; SEM, scanning electron microscopy; PI3K, phosphatidylinositol 3-kinase; AKT, protein kinase B; IRS, insulin receptor substrate; PIP3, phosphatidylinositol-(3,4,5)-trisphosphate; PDK1, 3-phosphoinositide-dependent protein kinase-1; GK, glucokinase; G6PC, glucose-6-phosphatase, catalytic; PEPCK, Phosphoenolpyruvate carboxykinase; GPC, glycerophosphocholine; PCho, phosphocholine; GSH, glutathione; GSSG, oxidized glutathione; BKS, normal control group; db-CON, model control group; db-SDF, model SDF group; ALT, glutamate pyruvate transaminase; AST, glutamic oxaloacetic transaminase; QC, quality control; RSD, relative standard deviation; PCA, principal component analysis; PLS-DA, partial least square discrimination analysis; OPLS-DA, orthogonal partial least square discrimination analysis; RPT, response permutation testing; VIP, variable importance in projection; ROS, reactive oxygen species.
