# Supplementary material for: Regulatory Effects of Functional Soluble Dietary Fiber from Saccharina japonica Byproduct on the Liver of Obese Mice with Type 2 Diabetes Mellitus
Source: Mar Drugs. 2022 Jan 21;20(2):91. doi: 10.3390/md20020091 (PMC8877147; doi:10.3390/md20020091)
Supplement: Supplementary file 1 [file marinedrugs-20-00091-s001.zip › Supplemental Table.pdf]

Supplemental Table S1. Primer sequences for qRT-PCR

Genes	Primers	Sequence (5′-3′)	NCBI Reference Sequence
IRS1	Forward	GAGTTGAGTTGGGCAGAATAGG	NC_000067.7
	Reverse	CCTATCTGCATGGTCATGTAGT	
PI3K	Forward	AAACAAAGCGGAGAACCTATTG	NC_000079.7
	Reverse	TAATGACGCAATGCTTGACTTC	
PDK1	Forward	GATAAGATCCTGCTTTTTCGGC	NC_000068.8
	Reverse	TCCTGGATATCACTTGCGATTT	
AKT	Forward	TCGATTATCTCAAACTCCTCGG	NC_000078.7
	Reverse	CGACTTCATCCTTTGCAATGAT	
GSK-3β	Forward	AGGAGAACCCAATGTTTCGTAT	NC_000082.7
	Reverse	ATCCCCTGGAAATATTGGTTGT	
GK	Forward	GCCCGAAAGAGTCTAAAGCG	NC_000086.8
	Reverse	AGCAGAGCCACGGTCATCA	
PEPCK	Forward	CGCAAGCTGAAGAAATATGACA	NC_000068.8
	Reverse	GATGACTGTCTTGCTTTCGATC	

β-actin	Forward	CTACCTCATGAAGATCCTGACC	NC_000071.7
	Reverse	CACAGCTTCTCTTTGATGTCAC	

Supplemental Table S2. Parameters for the assessment of these models (Type: the multivariate statistical analysis model was established, PRE: the number of principal components, ORT: the number of orthogonal components, N: the number of samples, R^2X (cum): the square of the percentage of row data retained in the X-axis direction, Cum: the cumulative result of several principal components, R^2Y: the square of the percentage of row data retained in the X-axis direction, Q^2 (cum): the cumulative prediction rate of the model, R^2 and Q^2: the parameter of the response sequencing test used to measure whether the model was overfitted)

group	type	PRE	ORT	N	R^2X (cum)	R^2Y (cum)	Q^2 (cum)	R^2	Q^2
db_SDF/db_CON	PCA	3	0	10	0.59				
db_SDF/db_CON	PLS	4	0	10	0.76	0.99	0.92		
db_SDF/db_CON	OPLS	1	3	10	0.76	0.99	0.86	0.97	-0.25

Supplemental Table S3. Significantly metabolite pathway enrichment (the rich factor was the ratio of number of different metabolites participating in this pathway and number of different metabolites participating in all pathways; *P*-value represented the significant difference of the metabolic pathway).

pathway name	rich factor	*p*-value	-lg (*p*-value)
choline metabolism in cancer	0.36	0.00	5.97

glycerophospholipid metabolism	0.12	0.00	5.61
bile secretion	0.05	0.00	3.03
pantothenate and CoA biosynthesis	0.11	0.00	2.88
cholesterol metabolism	0.20	0.00	2.57
ABC transporters	0.04	0.00	2.53
phospholipase D signaling pathway	0.18	0.00	2.49
protein digestion and absorption	0.06	0.01	2.23
neuroactive ligand-receptor interaction	0.06	0.01	2.10
pentose and glucuronate interconversions	0.05	0.01	2.04
retrograde endocannabinoid signaling	0.11	0.01	2.01
taurine and hypotaurine metabolism	0.09	0.01	1.89
linoleic acid metabolism	0.07	0.02	1.69
ferroptosis	0.07	0.02	1.66
huntington disease	0.33	0.02	1.62
central carbon metabolism in cancer	0.05	0.03	1.46
starch and sucrose metabolism	0.05	0.03	1.46
glutathione metabolism	0.05	0.04	1.44

glycerolipid metabolism	0.05	0.04	1.44
vitamin digestion and absorption	0.05	0.04	1.42
foxo signaling pathway	0.20	0.04	1.40
