# Supplementary figures and images for: Regulatory Effects of Functional Soluble Dietary Fiber from Saccharina japonica Byproduct on the Liver of Obese Mice with Type 2 Diabetes Mellitus
Source: Mar Drugs. 2022 Jan 21;20(2):91. doi: 10.3390/md20020091 (PMC8877147; doi:10.3390/md20020091)

# Correlation

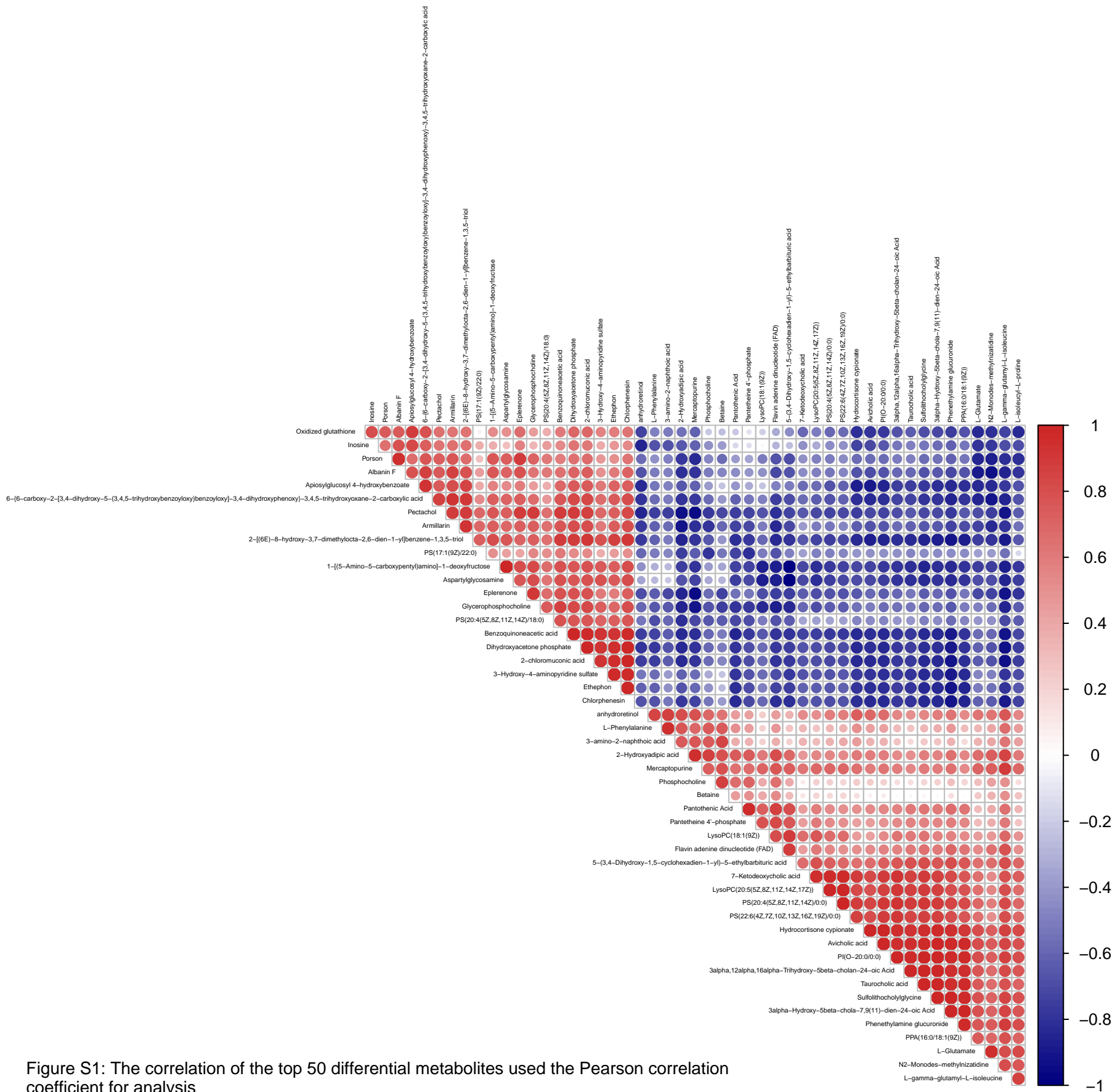

Supplement: Supplementary file 1 [file marinedrugs-20-00091-s001.zip › Supplemental Figure 1.pdf]
